# Detonation of fulminating gold produces heterogeneous gold nanoparticles

**DOI:** 10.1039/d3na01110k

**Published:** 2024-01-24

**Authors:** Jan Maurycy Uszko, Stephen J. Eichhorn, Avinash J. Patil, Simon R. Hall

**Affiliations:** a The Bristol Composites Institute (BCI), School of Civil, Aerospace and Design Engineering, University of Bristol, University Walk Bristol BS8 1TR UK; b School of Chemistry, University of Bristol Bristol BS8 1TS UK simon.hall@bristol.ac.uk

## Abstract

Fulminating gold, the first high-explosive compound to be discovered, disintegrates into a mysterious cloud of purple smoke, the nature of which has been speculated upon since its discovery in the 15th century. In this work, we show that the colour of the smoke is due to the presence of gold nanoparticles.

## Abstract 2

Nanoparticles are the solution to the mystery of unusual purple smoke created in the detonation of the first ever high explosive, fulminating gold.

## Graphical abstract

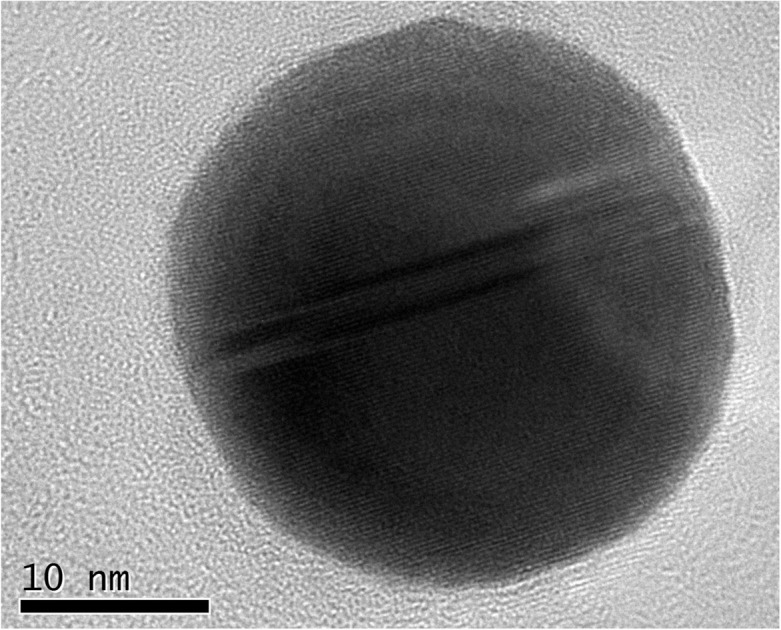

The alchemist's fascination with the transmutation of base metals into gold (chrysopoeia) led to the discovery of the first high-explosive compound, fulminating gold, an amorphous polymeric mixture of gold(iii), ammonia and chloride salts.^1^ Its first synthesis is dated to the 15th century, yet claims of initial discovery, publication and development of this material are complex and discussed in detail in an article by Wentrup in 2019.^2^

The process was described by Sebalt Schwärtzer and it initially required four to five days to complete, but since then, the process has been studied and improved to the point where fulminating gold can now be synthesized in minutes by simply mixing gold(iii) compounds with ammonia.^3^ The ease at which this material can be synthesized has stimulated research, leading to reviews of the current state of the art into fulminating gold being periodically published by scientific journals from “Über die Stickstoffverbindungen des Goldes” in 1915 to “Fulminating Gold and Silver” in 2019.^1,2,4^ Besides academic interest, the other motivator for research on fulminating gold has been safety. The danger of accidently creating highly explosive, touch-sensitive side products resulted in warnings on the use of gold(iii) compounds being printed in journals, editorial letters, chemical textbooks as far back as 1851, and safety manuals like the popular “Bretherick's Handbook of Reactive Chemical Hazards”.^1,5–7^ The interest in fulminating gold has even spread to digital media, with a video showing the synthesis of fulminating gold by Thomas De Prinse on his YouTube channel “Explosions&Fire” reaching almost 1 million views since it was first posted in 2020.^8^ In this video, De Prinse repeats a supposition that is often stated in relation to the source of the unusual red or purple colouration of the smoke created in the detonation of fulminating gold, that it is due to the presence of gold nanoparticles. There is circumstantial evidence that the smoke consists of gold nanoparticles, as it has been used to coat objects in a purple/crimson patina as described in “Opera Chymica” by Glauber,^2,9^ much in the same way that solutions of gold nanoparticles can be used to coat substrates with purple/red layers.^10^ Similar observations have been made in an analogous process described by Faraday in 1857, where a large current is passed through thin gold wire aerosolising metal, resulting in a similarly coloured cloud and coating.^11^ Analysis of the aerosol in the 1960s confirmed the presence of heterogenous gold nanoparticles with size depending on the voltage used to decompose the wire.^12^ Since then experiments have been repeated on wires of a range of metals, both in gases as well as in liquids, resulting in metal or metal oxide nanoparticles.^12–14^ To date, however, there has been no experimental verification of the composition of the smoke on the detonation of fulminating gold. Here, we show for the first time that the explosion of fulminating gold creates gold nanoparticles, ranging in size from 5 to over 300 nm. Furthermore, given the extreme heat and rapidity of their creation, they are more isotropic than nanoparticles created by conventional methods.

A typical synthesis was as follows: chloroauric acid (20 mg, Sigma-Aldrich) was dissolved in 1 ml of deionized water in a polypropylene tube. To this solution, ammonium hydroxide (28–30 w/w% Fisher Scientific) was added dropwise until an orange precipitate formed. The suspension was divided into four aliquots and each placed on separate aluminium foils to dry overnight in air at room temperature. After drying, samples of approximately 5 mg were detonated by the application of heat to the aluminium foil, whilst carbon-coated TEM grids (copper 200 mesh, Agar Scientific) were held above the foil to catch the resultant purple cloud. Fig. 1 shows a high-resolution TEM image of a single nanoparticle with visible lattice fringes, that have a spacing of 0.24 nm, consistent with the (111) crystal planes of Au. The selected area electron diffraction pattern illustrated in Fig. 2 and Table 1 confirms that they are indeed Au(0), as per the Joint Committee on Powder Diffraction Standards (JCPDS) card no. 04-0784.

**Fig. 1:**
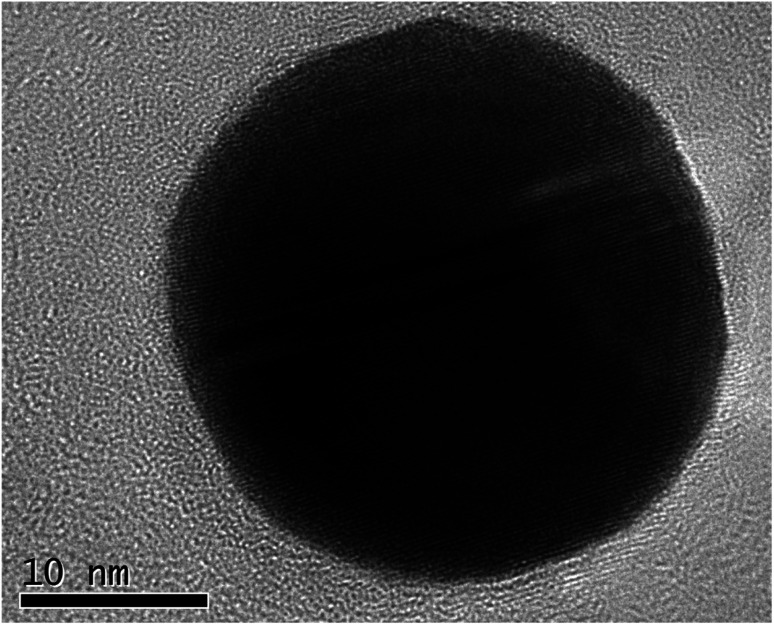
TEM image of a nanoparticle, from detonated fulminating gold, with visible lattice fringes.

**Fig. 2:**
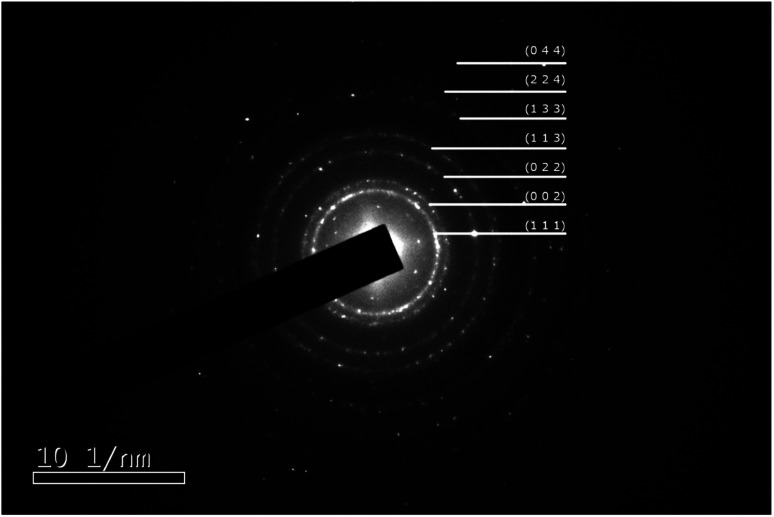
Selected area electron diffraction ring pattern from gold nanoparticles. The rings are indexed to Au as per the Joint Committee on Powder Diffraction Standards (JCPDS) card no. 04-0784.

**Table 1:** Indexed electron diffraction rings

Ring identification (JCPDS #04-0784)				
Plane	Radius [1/nm]		*d*-Spacing [nm]	
	Theor.	Measured	Theor.	Measured
(111)	4.245	4.072	0.236	0.246
(002)	4.902	4.668	0.204	0.214
(022)	6.932	6.705	0.144	0.149
(113)	8.129	7.847	0.123	0.127
(133)	10.684	10.380	0.094	0.096
(224)	12.007	11.522	0.083	0.087
(224)	12.007	12.217	0.083	0.082
(044)	13.865	13.956	0.072	0.072

TEM images of grids that were exposed to the purple cloud showed clusters of spherical nanoparticles exhibiting a wide size distribution from 5 nm to over 300 nm, with an average particle diameter of 40 nm [*σ* = 44 nm] illustrated in Fig. 3 and 4. The broad particle size distribution is indicative of the extreme rapidity and high temperature of the synthesis, with no possibility of achieving a lower polydispersity *via* the usual mechanisms where smaller particles are absorbed into larger ones (Ostwald ripening).^15,16^ The absence of well-defined facets in the nanoparticles is intriguing and further indicates the hot accelerated synthesis. The formation of the usual faceted or even triangular morphology of Au nanoparticles is effectively prevented through their creation by detonation. In this way, larger gold nanoparticles can be created with a sphericity more commonly seen in the early stages of formation when the nanoparticles are small.^16^ This work is proof of the long-supposed nature of the cloud produced on the detonation of fulminating gold, but also potentially opens the door to fast solvent- and capping agent-free syntheses of metal nanoparticles.

**Fig. 3:**
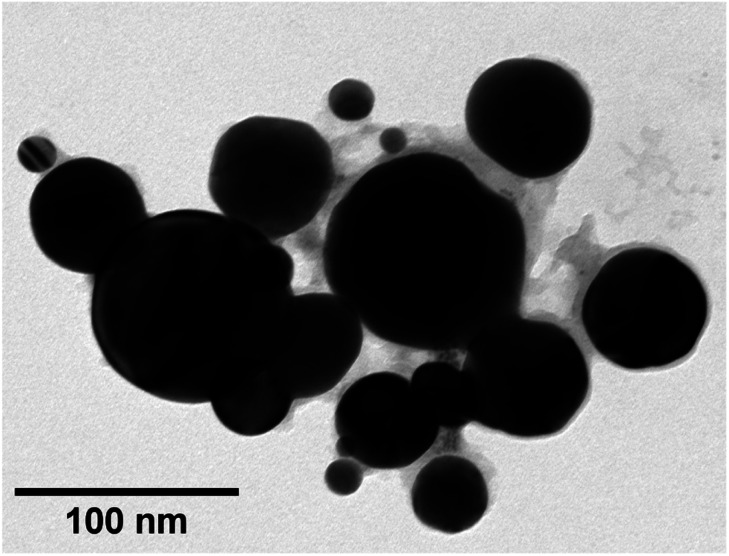
TEM image of a cluster of gold nanoparticles captured from detonated fulminating gold. The image demonstrates visually the broad dispersion of particle sizes.

**Fig. 4:**
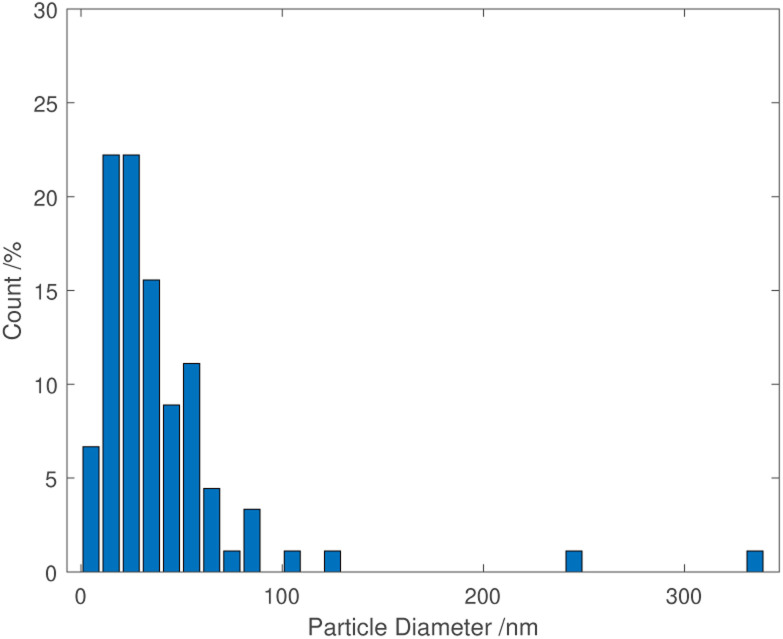
Particle size distribution of gold nanoparticles from the detonation of fulminating gold.

## Author contributions

Conceptualization: JMU, SRH. Methodology: JMU. Investigation: JMU. Visualization: JMU, SRH. Funding acquisition: SJE. Project administration: SRH, SJE, AJP. Supervision: SRH, SJE AJP. Writing – original draft: JMU, SRH. Writing – review & editing: JMU, SRH, SJE, AJP.

## Conflicts of interest

There are no conflicts to declare.

## Supplementary Material
